# Early Spontaneous Common Bile Duct Perforation

**DOI:** 10.7759/cureus.103292

**Published:** 2026-02-09

**Authors:** Eugenia Yoo, Alana M Hofmann, Ralph C Quillin, Christopher B Horn

**Affiliations:** 1 Family Medicine, University of Cincinnati College of Medicine, Cincinnati, USA; 2 Transplant Surgery, Willis Knighton John C. McDonald Transplant Center, Shreveport, USA; 3 Transplant Surgery, University of Cincinnati College of Medicine, Cincinnati, USA; 4 Surgery, University of Cincinnati Medical Center, Cincinnati, USA; 5 Center For Sustainment of Trauma and Readiness Skills, United States Air Force, Cincinnati, USA

**Keywords:** advanced endoscopy, biliary diseases, case report, cholecystitis, common bile duct perforation, hepatobiliary, minimally invasive surgery

## Abstract

Non-iatrogenic, non-traumatic common bile duct (CBD) perforation is a rare finding in the adult population and is more commonly reported in the pediatric population. Etiologies of CBD perforation in adults include traumatic injury, increased intraductal pressure due to choledocholithiasis, and biliary duct necrosis from underlying inflammation such as chronic pancreatitis. We present the case of a 59-year-old woman who initially presented with acute perforated cholecystitis with pancreatitis with intra-operative findings of a CBD perforation, which is not well-described in the literature. Management included endoscopic retrograde cholangiopancreatography with CBD stent placement and delayed cholecystectomy. This case is notable for the short period of symptoms prior to perforation and highlights the need for a higher degree of suspicion for alternative diagnoses when confronted with perforated cholecystitis.

## Introduction

Common bile duct (CBD) perforations are rare in adults, with trauma and iatrogenic injuries being frequent causes. Reported etiologies of non-traumatic, non-iatrogenic CBD perforations include perforation from increased intraductal pressure due to stones in the CBD, necrosis from underlying inflammatory processes, such as infection, and chronic pancreatitis [1-16]. There are limited reports of early non-traumatic, non-iatrogenic CBD perforation due to acute pancreatitis or acute cholecystitis. To our knowledge, only four cases have been previously described [5,7,8,10]. In these cases, perforation occurred after one to four days of symptom onset in patients ranging from 3 to 71 years. In three of these cases, the patients were managed with cholecystectomy and repair of the CBD over a T-tube [5,7,8]. The final case was managed with endoscopic retrograde cholangiopancreatography (ERCP) stenting alone, and the patient was lost to follow-up prior to cholecystectomy [10].

We present the case of an otherwise healthy patient who presented with spontaneous CBD perforation after two days of symptomatology managed with ERCP stenting and interval cholecystectomy. 

## Case presentation

**Figure 1:**
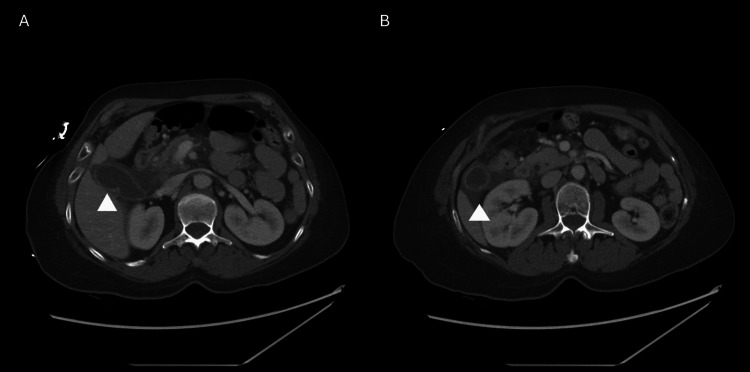
Representative CT images demonstrating (A) gallbladder wall thickening consistent with acute cholecystitis (arrow) and (B) free pericholecystic fluid consistent with perforated cholecystitis

A 59-year-old woman without past medical history presented to the Emergency Department with complaints of two days of epigastric pain radiating to the right upper quadrant associated with nausea, emesis, and chills. She reported no prior episodes of abdominal pain and had no prior Emergency Department visits for abdominal pain. Vital signs were within normal limits. Physical examination revealed a soft, non-distended abdomen with epigastric and right upper quadrant tenderness to palpation without guarding. Laboratory findings included leukocytosis (WBC: 16,200/mm^3^; reference range:3.8-10.8) and increased liver enzymes (alkaline phosphatase 309 U/L; reference range: 36-125, AST 497 U/L; reference range: 13-39, ALT 328 U/L; reference range: 7-52) and lipase (2,288 U/L; reference range: 4-82). A computed tomography (CT) scan of the abdomen and pelvis, shown in Figure 1, showed acute perforated cholecystitis with right paracolic gutter and rectouterine pouch calculi.

Ultrasound of the abdomen showed a 4 mm CBD and no intra- or extrahepatic ductal dilation. The gallbladder was thickened up to 1.3 cm and mildly distended with small stones and sludge in the lumen. These findings raised a concern for perforated cholecystitis in the setting of acute pancreatitis.

**Figure 2:**
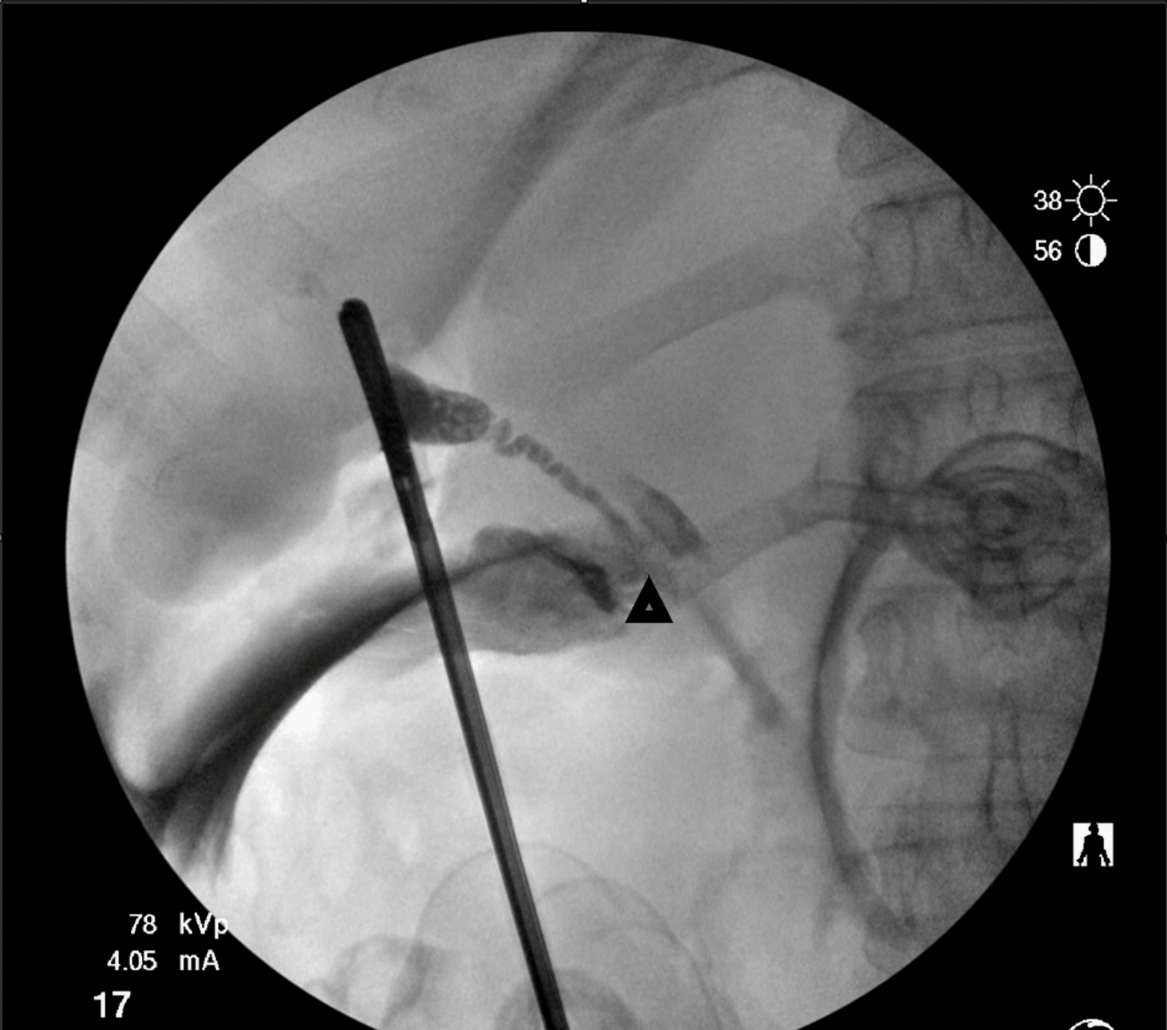
Intraoperative cholangiogram during index operation demonstrating contrast extravasation from common bile duct perforation (arrow)

The patient was initially started on intravenous piperacillin and tazobactam. Two days later with improvement in her pancreatitis, she was taken to the operating room for laparoscopic cholecystectomy with possible intraoperative cholangiogram. Upon entering the abdomen, there was significant bile spillage. A single epigastric port was placed under direct visualization and the bile was suctioned free with a suction irrigator. Two additional right subcostal ports were placed. The gall bladder was grasped and elevated. No obvious perforation could be identified in the gallbladder, but bile was noted to be flowing from the hepatoduodenal ligament prior to completion of any dissection. This raised suspicion of a perforated CBD. The gallbladder was dissected free, and a critical view of safety was obtained. An intraoperative cholangiogram, seen in Figure 2, was performed, which showed extravasation from the CBD without retrograde filling of hepatic ducts or filling of the duodenum. 

With these findings, the patient was transferred to a tertiary center with hepatopancreatic biliary surgeons and transplant surgery for continued management. Upon transfer, an ERCP confirmed extravasation of injected contrast from the mid-CBD near the suspected cystic duct origin. The common hepatic duct, biliary confluence, and bilateral intrahepatic ducts appeared normal. A biliary sphincterotomy was performed and two 7 Fr x 9 cm double-pigtail stents and one 7 Fr x 10 cm Cotton-Leung stent were placed across the site of leak. The patient was discharged on a seven-day course of oral amoxicillin-clavulanate.

**Figure 3:**
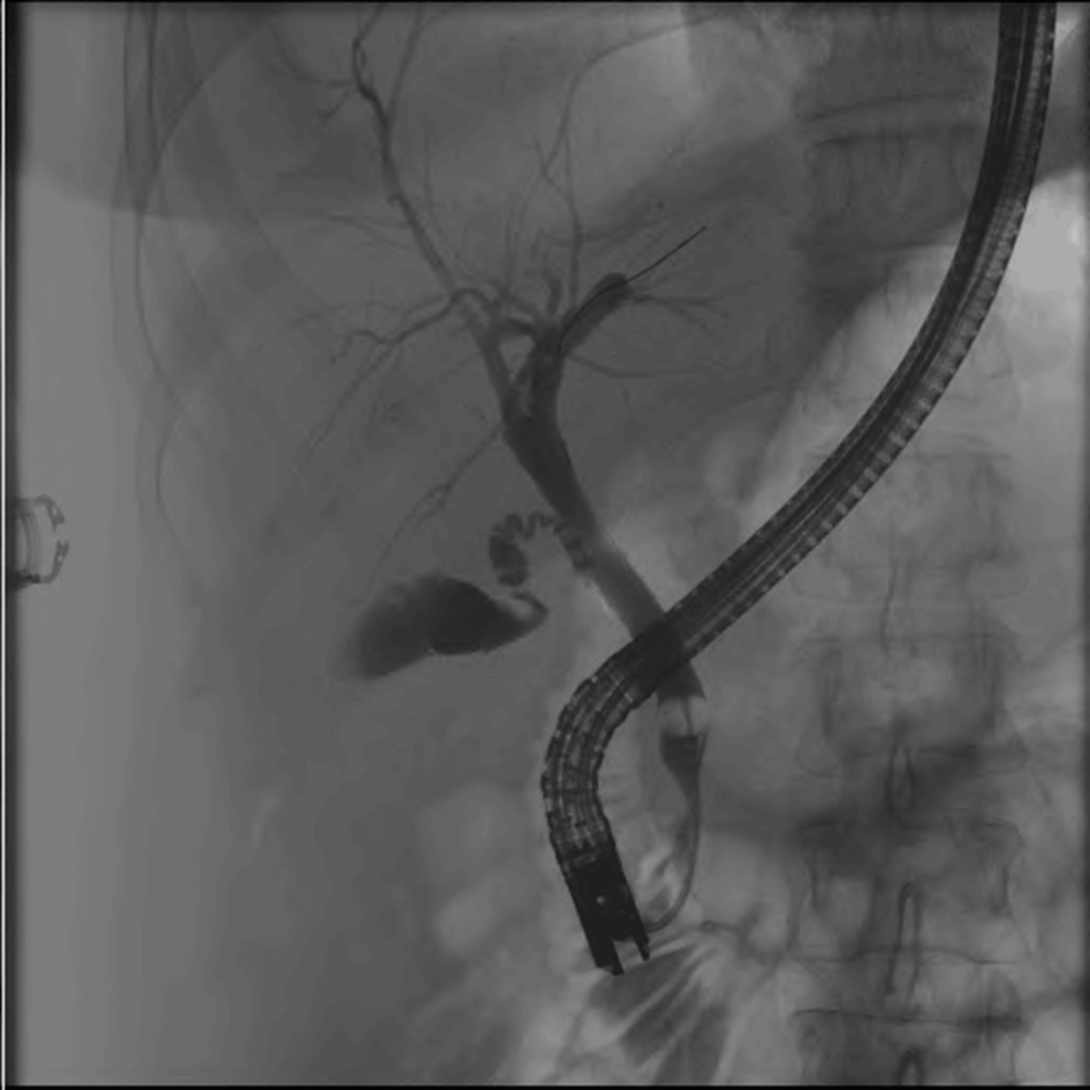
Endoscopic retrograde cholangiogram demonstrating resolution of common bile duct perforation prior to completion of cholecystectomy

In the outpatient setting, a repeat CT revealed a new intrahepatic collection. A second ERCP, completed six weeks after stent placement, seen in Figure 3, revealed mild mid-common duct stenosis without upstream dilation, and normal common hepatic and bilateral intrahepatic ducts with a patent cystic duct. No contrast extravasation was seen from the CBD or within the liver. The prior stents were replaced with six new plastic biliary stents. Four months after the second ERCP, these six stents were removed, and a repeat cholangiogram showed a normal CBD, cystic duct, and bilateral intrahepatic ducts. 

Three months following the removal of stents, the patient underwent laparoscopic cholecystectomy. The intraoperative course was notable for the presence of scar tissue and fused cystic artery and cystic duct from the prior operation, but otherwise without complications. Surgical pathology showed chronic cholecystitis and cholelithiasis without malignancy. Two weeks after the operation, the patient was seen for a postoperative outpatient follow-up visit and found to be doing well with resolution of her symptoms.

## Discussion

Non-traumatic, non-iatrogenic CBD perforation is rare, and more commonly encountered in the pediatric population than in the adult population [1-5,7,8,11]. The most common site of non-traumatic, non-iatrogenic CBD perforation in adults is the junction of the cystic and hepatic duct [7,8].

Prior reports of non-traumatic, non-iatrogenic CBD perforation suggest that the most common causes of perforation are acquired weaknesses in the wall of the bile duct or increases in intraductal pressure [8]. The proposed etiologies of bile duct weakness include chronic inflammation, congenital malformations such as choledochal cysts, or ischemia [6-16]. Increased intraductal pressure is generally due to acquired distal obstruction, caused by biliary stones, tumors, or congenital stenosis of the Ampulla of Vater [6-16].

With chronic progression of inflammation or obstruction, CBD perforation is significantly more common than perforations early in the disease course. To our knowledge, there has been one prior report of spontaneous CBD perforation due to acute pancreatitis, which occurred five days after symptom onset [15]. The authors propose that regurgitation of activated pancreatic enzymes from the pancreatic duct may have contributed to the bile duct perforation. 

In our case, CBD perforation developed within two days of symptom onset in the setting of acute pancreatitis and cholecystitis. Given the short duration of symptoms, it is unlikely that the perforation developed due to prolonged ischemia from external compression (Mirizzi's syndrome) or from choledocholithiasis. Notably, CBD perforation was initially misdiagnosed on CT. While magnetic resonance cholangiopancreatography (MRCP) may have higher sensitivity for CBD perforation, the cost of MRCP and time required to obtain an MRCP likely limit its applicability. As such, MRCP should be limited to those cases with high clinical suspicion for this rare disease. 

## Conclusions

This case is unique in the timing from symptom onset to perforation as well as the excellent outcome of the patient using minimally invasive means. Due to the rarity and difficulty of detecting spontaneous CBD perforation on CT imaging as well as the variability of timing of perforation after symptom onset, CBD perforation may initially be mistaken for perforated cholecystitis. A high index of suspicion and knowledge of this condition can aid in early diagnosis and management to prevent morbidity, especially with the aid of an intraoperative cholangiogram. Our case was confounded by imaging consistent with acute cholecystitis, resulting in an initial misdiagnosis of perforated cholecystitis rather than a perforation of the CBD. Despite this initial misdiagnosis, an intraoperative cholangiogram demonstrated a spontaneous perforation of the CBD, which was managed with a multidisciplinary team, with an excellent outcome for the patient.
